# Age-Dependent Anti-migraine Effects of Valproic Acid and Topiramate in Rats

**DOI:** 10.3389/fphar.2018.01095

**Published:** 2018-09-25

**Authors:** Pokai Huang, Ping-Hung Kuo, Ming Tatt Lee, Lih-Chu Chiou, Pi-Chuan Fan

**Affiliations:** ^1^Department of Pediatrics, E-da Dachang Hospital, Kaohsiung, Taiwan; ^2^Department of Internal Medicine, National Taiwan University Hospital, College of Medicine, National Taiwan University, Taipei, Taiwan; ^3^Graduate Institute of Brain and Mind Sciences, College of Medicine, National Taiwan University, Taipei, Taiwan; ^4^Graduate Institute of Pharmacology, College of Medicine, National Taiwan University, Taipei, Taiwan; ^5^Faculty of Pharmaceutical Sciences, UCSI University, Kuala Lumpur, Malaysia; ^6^Graduate Institute of Acupuncture Science, China Medical University, Taichung, Taiwan; ^7^Department of Pediatrics, National Taiwan University Hospital, College of Medicine, National Taiwan University, Taipei, Taiwan

**Keywords:** valproic acid, topiramate, age difference, migraine, CGRP

## Abstract

**Background:** Valproic acid (VPA) and topiramate (TPM), initially developed as antiepileptics, are approved for migraine prophylaxis in adults but not children. The differences in their antimigraine mechanism(s) by age remain unclear.

**Methods:** A migraine model induced by intra-cisternal (*i.c.*) capsaicin instillation in pediatric (4–5 weeks) and adult (8–9 weeks) rats was pretreated with VPA (30, 100 mg/kg) or TPM (10, 30, 100 mg/kg). Noxious meningeal stimulation by the irritant capsaicin triggered trigeminovascular system (TGVS) activation mimicking migraine condition, which were assessed peripherally by the depletion of calcitonin gene-related peptide (CGRP) in sensory nerve fibers of the dura mater, the increased CGRP immunoreactivity at trigeminal ganglia (TG) and centrally by the number of c-Fos-immunoreactive (c-Fos-ir) neurons in the trigeminocervical complex (TCC). Peripherally, CGRP released from dural sensory nerve terminals of TG triggered pain signal transmission in the primary afferent of trigeminal nerve, which in turn caused central sensitization of the TGVS due to TCC activation and hence contributed to migraine.

**Results:** In the VPA-treated group, the central responsiveness expressed by reducing the number of c-Fos-ir neurons, which had been increased by *i.c.* capsaicin, was significant in pediatric, but not adult, rats. Inversely, VPA was effective in peripheral inhibition of elevated CGRP immunoreactivity in the TG and CGRP depletion in the dura mater of adult, but not pediatric, rats. In TPM group, the central responsiveness was significant in both adult and pediatric groups. Peripherally, TPM significantly inhibited capsaicin-induced CGRP expression of TG in adult, but not pediatric, rats. Interestingly, the capsaicin-induced depletion of CGRP in dura was significantly rescued by TPM at high doses in adults, but at low dose in pediatric group.

**Conclusion:** These results suggest VPA exerted peripheral inhibition in adult, but central suppression in pediatric migraine-rats. In contrast, TPM involves both central and peripheral inhibition of migraine with an optimal therapeutic window in both ages. These findings may clarify the age-dependent anti-migraine mechanism of VPA and TPM, which may guide the development of new pediatric anti-migraine drugs in the future.

## Introduction

As compared with adult migraine (12–17%) (Stewart et al., 1994; Wang, 2003), pediatric migraine is characterized by more bilateral involvement, shorter duration, less severity, better response to treatment (Swaiman et al., 1999), and lower prevalence (6–11%) (Abu-Arefeh and Russell, 1994; Wang et al., 2005). However, the mechanism(s) contributing to these differences remain unclear. The trigemino-vascular system (TGVS) consists of dural blood vessels which are innervated by unmyelinated small trigeminal afferent axons, trigeminal ganglia (TG), and the trigemino-cervical complex (TCC) (Liu-Chen et al., 1983; Mayberg et al., 1984; Uddman et al., 1985; Tsai et al., 1988; Nozaki et al., 1990). Peripheral sensitization of the TVGS via chemical stimulation may trigger migraine-like symptoms (Moskowitz, 1984, 1991). Central sensitization of the TGVS by TCC activation, which is involved in both nociceptive processing and cerebrovascular regulation, also plays a pivotal role in migraine pathophysiology (Lance et al., 1983). Activation of trigeminal nerves causes initial release of CGRP, which is regulated by antimigraine drugs (Durham and Russo, 1999). In a rat migraine model induced by intra-cisternal (*i.c.*) instillation of capsaicin, we have compared differences in central and peripheral responsiveness of the TGVS between pediatric and adult rats (Fan et al., 2012). We found that pediatric rats had a comparable central responsiveness, measured by the number of activated TCC neurons, as adult rats, but displayed less peripheral responsiveness than adults, including the CGRP immunoreactivity (CGRP-ir) in the TG and CGRP depletion (inversely reflected by CGRP-ir) in the dura mater (Fan et al., 2012).

Valproic acid (VPA) and topiramate (TPM), both clinically effective in migraine prophylaxis were used to validate this animal model. Both developed as antiepileptic drugs (AEDs), they are widely used to treat a number of non-epileptic pathologies, including headache prophylaxis, due to their neuronal stabilizing effects. The hypothesis of cortical hyperexcitability in migraine pathogenesis has led to a great number of large, randomized clinical trials to evaluate the efficacy and safety of AEDs in headache prevention (Colombo et al., 2008). However, it is not clear why only a few AEDs have a good efficacy in migraine prophylaxis. To stabilize the hyperexcitability in the cortex may not be the only mechanism for those effective epileptic drugs in migraine prophylaxis. The stabilization of TGVS activity, in terms of central and peripheral responses (Fan et al., 2012), by those AEDs may also play a role in their anti-migraine actions.

Among the AEDs, only VPA and TPM are approved by the Food and Drug Administration (FDA) for migraine prevention in adults and recently TPM was approved in pediatric migraine, but not VPA. However, their off-label use for pediatric migraine is a common practice. VPA is a GABA transaminase inhibitor and activator of glutamic acid decarboxylase, a GABA synthesizing enzyme (Chapman et al., 1982; Rogawski and Loscher, 2004). Thus, it can enhance GABAergic transmission, which may lead to the reduction of neurogenic inflammation response, contributing to its anti-migraine effect (Cutrer et al., 1997). However, its effect on the CGRP mediated response of TGVS has not yet been studied before. TPM has been demonstrated to modify several receptor- and voltage-gated ion channels, including Na^+^ channels, Ca^2+^ channels, and non-NMDA receptors, and GABA_A_ receptors which are involved in the initiation and propagation of seizure activity (White, 2005). Several animal studies have reported that TPM can reduce the TGVS activity. It reduced superior sagittal sinus-evoked firing of TCC neurons in a cat model of migraine (Storer and Goadsby, 2004), attenuated neurogenic dural vasodilation probably through inhibiting the release of CGRP from prejunctional trigeminal neurons (Akerman and Goadsby, 2005a) and inhibited regional cerebral blood flow changes and the initiation and propagation of cortical spreading depression in rats and cats (Akerman and Goadsby, 2005b). In trigeminal neuronal culture, TPM decreased CGRP release induced by KCl, nitric oxide and proton in a time- and concentration-dependent manner without altering the amount of unstimulated CGRP release (Durham, 2006; Durham et al., 2006). These animal studies suggest TPM can decrease the peripheral responsiveness of the TGVS. However, there are few studies comparing its effect on the central and peripheral responsiveness simultaneously in animals.

In this study, to better understand the age differences in antimigraine mechanism(s) of VPA and TPM, we examined central and peripheral effects of these two drugs on the same animal model of migraine induced by *i.c.* capsaicin instillation (Fan et al., 2012) and compared the age-dependency in their effects using pediatric and adult rats.

## Materials and Methods

### Animals

All animal care and experimental protocols were approved by the Institution of Care and Use of Laboratory Animals of the College of Medicine of National Taiwan University. The age for weaning in rats is P21 (3 weeks old), equal to 2 years in humans whereas sexual maturity is achieved after 7 weeks. Therefore, male Wistar rats at 8–9 weeks (275–300 g, adult group) and 4–5 weeks (101–125 g, pediatric group) were used. They were housed in an animal room with a 12-h light/12-h dark cycle and free access to food and water.

### Intra-Cisternal Instillation of Capsaicin

The migraine model induced by *i.c.* instillation of capsaicin is similar to our previous study (Fan et al., 2012). Briefly, the rat received chloral hydrate (400 and 100 mg/kg, *i.p.*, respectively, for inducing and maintaining) as anesthesia and catheterized with a catheter (PE-10, SIMS Portex Ltd, Hythe, United Kingdom) inserted 3 mm deep into the cisterna magna, followed by being placed in a prone position for 5.5 h. Rats received *i.v.* injection of VPA (Sanofi-Aventis, Paris, France) or its vehicle, or *i.p.* injection of TPM (Sigma-Aldrich, St. Louis, MO, United States) or its vehicle 30 min before capsaicin administration. Then, the capsaicin solution (10 nmol, 100 μl) was instilled through the catheter into the cisterna magna over 1 min. The rat was then placed in a reverse Trendelenburg position (-30°) for 30 min in order to facilitate capsaicin distribution within the subarachnoid space, followed by a prone position for another 90 min. Capsaicin (Sigma Chemical, St. Louis, MO, United States) was dissolved in the vehicle solution containing 10% ethanol and 10% Tween 80, and sonicated for 5 min, and then further diluted (1:100) in artificial CSF as a stock solution stored at 4°C. For the sham control group, 100 μl of the vehicle was administered by *i.c.* instillation. Two hours after capsaicin instillation, the rat was euthanized by an overdose of chloral hydrate and then perfused via the ascending aorta with paraformaldehyde (4%) as described previously (Fan et al., 2012).

### Drug Administration of VPA and TPM

VPA and TPM were administered at three doses in a 1X, 3X, 10X design applied in 4 animals in each of the groups with pretreatment. The doses of VPA (*i.v.*) injected was 30, 100, and 300 mg/kg (Cutrer et al., 1995b). However, the mortality rate was very high at 300 mg/kg. Thus, the results from the VPA groups at the doses of 30 and 100 mg/kg were demonstrated. TPM (*i.p.*) was instilled at the doses of 10, 30, and 100 mg/kg according to previous studies (Akerman and Goadsby, 2005a,b).

### TCC Brain Sections

The brainstem with attached cervical cord was dissected, stored overnight, dehydrated and then were serially sectioned (50 μm) using a cryostat (LEICA CM3050S, Nussloch, Germany) from 1 mm rostral to the obex to the C6 level of the spinal cord. The sections at +0.6, -1.2, and -9 mm from the obex of adult and +0.6, -0.6, and -6 mm from the obex of pediatrics (Fan et al., 2012) were collected and subjected to c-Fos immunohistochemical staining. Four rats in each group were studied and the total number of c-Fos-immunoreactive (c-Fos-ir) TCC neurons was estimated based on the formula derived in our previous study (Fan et al., 2012).

### TG Slice Sections and Quantification

The rat TG was dissected, stored overnight, embedded by paraffin wax and then serially sectioned at 5 μm thickness using a microtome (LEICA RM2245, Nussloch, Germany). Every third section was used for CGRP immunohistochemical staining, and we analyzed nine sections per ganglion in the TG. The ratio of positivity was defined as positive-stained area divided by total section area. The strength of immunoreactivity was measured by mean staining intensity subtracted from background using the software of image J. The CGRP-ir of TG was quantified as ratio of positivity multiplied by strength of immunoreactivity.

### Dura Mater Preparations and Quantification

The dura mater dissected from the cranial cavity of the rat was prepared as described previously (Fan et al., 2012) for CGRP immunohistochemical staining. Four identical areas of fixed regions of the dura were selected and viewed under an inverted microscope (ZEISS Axio Observer.D1). The total lengths of positive-stained segmented lines in the field (100X) were measured in pixels by the software of image J. The CGRP density of dura was quantified and averaged as length (pixels) per field.

### Immunohistochemistry of c-Fos Protein in TCC Sections

Free-floating immunohistochemistry of c-Fos protein was conducted using the avidin-biotin method as described previously (Fan et al., 2012), incubated with an anti-c-Fos rabbit polyclonal antibody (Calbiochem, San Diego, CA, United States) in 1:7000 dilution at 4°C for 48 h followed by incubation with biotinylated anti-rabbit IgG (Vector Labs, Burlingame, CA, United States) in 1:200 dilution for 2 h at room temperature and then incubated with horseradish peroxidase avidin D (Vector Labs, Burlingame, CA, United States) in 1:500 dilution for 1 h in the dark at room temperature. Immunoreactions were visualized using the DAB Reagent kit (KPL, Gaithersburg, MD, United States).

c-Fos-ir neurons, i.e., neurons with stained nuclei, were counted under a microscope (Olympus BX51, Essex, United Kingdom) by an observer blinded to the age group. Data were confirmed in randomly selected sections by a second investigator who was also blinded to the age group.

### Immunohistochemistry of CGRP in TG Slices and Dura Mater

Immunohistochemical staining of CGRP was performed with the avidin-biotin method with a protocol similar to that for c-Fos staining except using a 30 min-blocking incubation, anti-CGRP rabbit polyclonal antibody (1:1000, Calbiochem, San Diego, CA, United States), and horseradish peroxidase avidin D (1:200 Vector Labs, Burlingame, CA, United States).

### Statistical Analysis

All analysis was done using IBM SPSS Statistics 20 for Windows. Data were expressed as mean ± SE unless otherwise specified. Each treatment group was performed individually. Differences between the drug (VPA or TPM) treatment group and capsaicin only group were compared using the Mann–Whitney *U*-test. Statistical significance was a *p*-value < 0.05.

## Results

### VPA Reduced the Number of Capsaicin Induced c-Fos-ir TCC Neurons in Pediatric but Not Adult Rats

**FIGURE 1:**
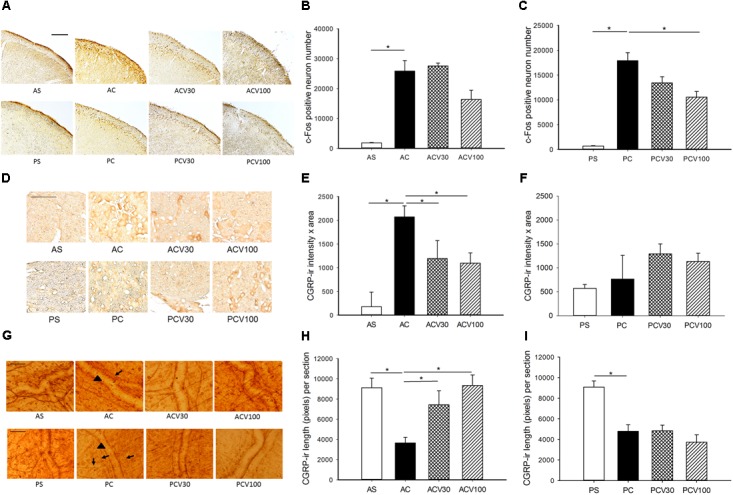
c-Fos-ir in trigeminocervical complex (TCC) and CGRP-ir in trigeminal ganglia (TG) and dura of adult and pediatric rats treated with valproic acid (VPA). **(A)** The immunochemical stain of c-Fos shows c-Fos-ir at various dosages in both age groups. **(B)** Intracisternal capsaicin (*i.c.*) significantly induced c-Fos-ir in TCC of the adult compared to sham (AC *vs.* AS, *p* = 0.021). The capsaicin induced c-Fos-ir is not significantly suppressed by VPA in adult at either 30 (ACV30 vs. AC) or 100 (ACV100 vs. AC) mg/kg. **(C)** Capsaicin *i.c.* significantly induced c-Fos-ir in TCC of the pediatric (PC vs. PS, *p* = 0.021). The capsaicin induced c-Fos-ir is significantly suppressed by VPA in pediatric at 100 mg/kg (PCV100 vs. PC, *p* = 0.021) but not 30 mg/kg (PCV30 vs. PC). **(D)** The immunochemical stain of TG shows CGRP-ir at various dosages in both age groups. **(E)** Capsaicin *i.c.* significantly induced CGRP-ir in TG of the adult (AC vs. AS, *p* = 0.021). The capsaicin induced CGRP-ir of TG is suppressed by VPA in the adult group, borderline at 30 mg/kg (ACV30 vs. AC, *p* = 0.05), and significant at 100 mg/kg (ACV100 vs. AC, *p* = 0.034). **(F)** The capsaicin induced CGRP-ir is not increased compared to sham in pediatric group (PC vs. PS). H-stat, *p* > 0.05. **(G)** The immunochemical stain of dura shows linear CGRP-ir (arrow) along the meningeal vessels (arrowhead) at various dosages of VPA in both age groups. **(H)** Capsaicin *i.c.* significantly depletes CGRP-ir in the dura of adult (AC vs. AS, *p* = 0.034). The depletion of CGRP is significantly reversed by VPA at 30 mg/kg (ACV30 vs. AC, *p* = 0.034) and 100 mg/kg (ACV100 vs. AC, *p* = 0.014) in adult group. **(I)** Capsaicin *i.c.* significantly depletes CGRP-ir in the dura of pediatric (PC vs. PS, *p* = 0.021). The rescue of CGRP depletion is not obvious at 30 (PCV30 vs. PC) and 100 mg/kg (PCV100 vs. PC) in pediatric group. *n* = 4 in each group, ^∗^*p* < 0.05 by Mann–Whitney *U*-test, scale bar: 100 μm.

After rats receiving *i.c.* capsaicin (10 nmol) instillation for 2 h, the number of c-Fos-ir TCC neurons was significantly increased in both adult (**Figure 1A**) and pediatric (**Figure 1B**) rats, as reported in our previous study (Fan et al., 2012). In the group pretreated with VPA at 100 mg/kg (*i.v.*), but not 30 mg/kg, the number of c-Fos-ir TCC neurons was decreased significantly in pediatric rats (*p* = 0.021) (**Figure 1B**), but not in adult rats at either 30 or 100 mg/kg (**Figure 1A**). This suggests that the central responsiveness, expressed by c-Fos-ir neurons in TCC, to VPA is only significant at a high dose in pediatric rats, but not in adult rats even at high does.

### VPA Reduced Elevated TG CGRP-ir and Dural CGRP Depletion in Adult but Not Pediatric Rats

After *i.c.* capsaicin instillation, the CGRP-ir of TG was significantly increased in adult (*p* = 0.021) (**Figure 1E**) but not pediatric (**Figure 1F**) rats, as reported in our previous study (Fan et al., 2012). In the adult group pretreated with VPA (*i.v.*), the CGRP-ir of TG was significantly decreased at 100 mg/kg (*p* = 0.034) and borderline at 30 mg/kg (*p* = 0.05) (**Figure 1E**). VPA was effective in peripheral inhibition of CGRP expression in the TG of adult rats in a dose dependent way (**Figure 1E**).

After *i.c.* capsaicin instillation, the depletion of CGRP in dura was quantified by measuring CGRP-ir length in this study and was significant in both adult (*p* = 0.034) (**Figure 1H**) and pediatric (*p* = 0.021) (**Figure 1I**) rats. The depletion of CGRP was significantly reversed by VPA at 30 mg/kg (*p* = 0.034) and 100 mg/kg (*p* = 0.014) in adult group (**Figure 1H**), but not obviously at 30 and 100 mg/kg in pediatric group (**Figure 1I**). VPA was effective in peripheral rescue of capsaicin induced CGRP depletion in the dura of adult rats in a dose dependent way, but not in pediatric rats.

These results that VPA is effective in treating capsaicin induced nociception in both age groups validate this animal model. It suggests the anti-migraine mechanism of VPA involves mainly peripheral inhibition of TGVS in adult rats, whereas central suppression of TCC in pediatrics. VPA at high dose, 100 mg/kg, is better than low dose, 30 mg/kg, in both central and peripheral responsiveness in this migraine model.

### TPM Reduced c-Fos-ir TCC Neurons in Both Age Groups

**FIGURE 2:**
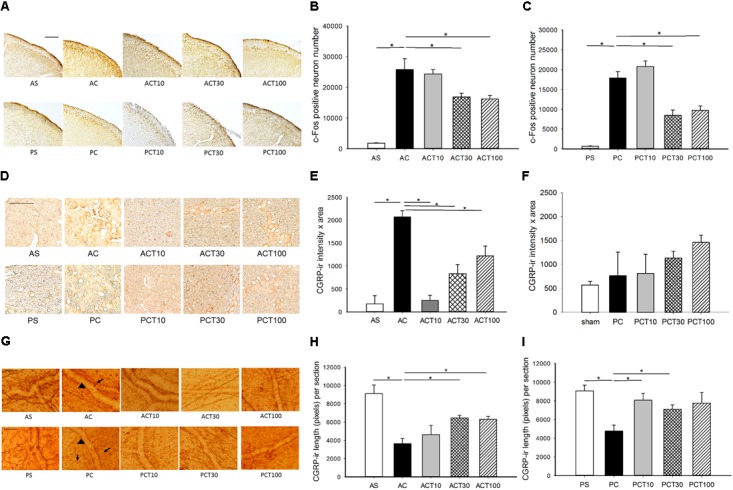
c-Fos-ir in trigeminocervical complex (TCC) and CGRP-ir in trigeminal ganglia (TG) and dura of adult and pediatric rats treated with topiramate (TPM). **(A)** The immunochemical stain of c-Fos shows c-Fos-ir at various dosages of TPM in both age groups. **(B)** Intracisternal capsaicin (*i.c.*) significantly induced c-Fos-ir in TCC of the adult compared to sham (AC vs. AS, *p* = 0.021). The capsaicin induced c-Fos-ir is significantly suppressed by TPM in adult at either 30 (ACT30 vs. AC, *p* = 0.043) or 100 (ACT100 vs. AC, *p* = 0.021) mg/kg, but not 10 mg/kg (ACT10 vs. AC). **(C)** Capsaicin significantly induced c-Fos-ir in TCC of the pediatric (PC vs. PS, *p* = 0.021). The capsaicin induced c-Fos-ir is significantly suppressed by TPM in pediatric at either 30 (PCT30 vs. AC, *p* = 0.021) or 100 (ACT100 vs. AC, *p* = 0.021) mg/kg, but not 10 mg/kg (PCT10 vs. AC). **(D)** The immunochemical stain of TG shows CGRP-ir at various dosages of TPM in both age groups. **(E)** Capsaicin significantly induced CGRP-ir in TG of the adult (AC vs. AS, *p* = 0.046). The capsaicin induced CGRP-ir of TG is significantly suppressed by TPM in the adult group at all the three doses (ACT vs. AC, *p* < 0.05) in a dose dependent way. **(F)** The capsaicin induced CGRP-ir is not increased compared to sham in pediatric group (PC vs. PS). H-stat, *p* > 0.05. **(G)** The immunochemical stain of dura shows linear CGRP-ir (arrow) along the meningeal vessels (arrowhead) at various dosages of TPM in both age groups. **(H)** Capsaicin significantly depletes CGRP-ir in the dura of adult (AC vs. AS, *p* = 0.034). The depletion of CGRP is significantly reversed by TPM at 30 mg/kg (ACT30 vs. AC, *p* = 0.021) and 100 mg/kg (ACT100 vs. AC, *p* = 0.021) in adult group. **(I)** Capsaicin *i.c.* significantly depletes CGRP-ir in the dura of pediatric (PC vs. PS, *p* = 0.028). The rescue of CGRP depletion is significant at 10 mg/kg (PCT10 vs. PC, *p* = 0.029) and at 30 (PCT30 vs. PC, *p* = 0.043) but not obvious at 100 mg/kg (PCT100 vs. PC, *p* = 0.08) in the pediatric group. *n* = 4 in each group, ^∗^*p* < 0.05 by Mann–Whitney *U*-test, scale bar: 100 μm. For illustrative purpose, immunohistograms of the sham and control groups (AS, AC, PS, and PC) are the same as used in **Figure 1**.

Topiramate reduced the number of capsaicin induced c-Fos-ir TCC neurons at 30 and 100 mg/kg in both adult (*p* = 0.043 and 0.021, respectively) (**Figures 2A,B**) and pediatric (*p* = 0.021) (**Figures 2A,C**), but not at 10 mg/kg (*p* > 0.05) in both age groups (**Figures 2B,C**). The central responsiveness to TPM in both age groups is significant at the dose of 30 mg/kg or higher.

### TPM Reduced TG CGRP-ir in Adult and Rescued Dura CGRP Depletion in Both Age Groups

Topiramate significantly reduced capsaicin-elevated CGRP-ir in TG at 10, 30, and 100 mg/kg (*p* < 0.05) in a dose-dependent way with a better response at a lower dose in adult rats (**Figures 2D,E**). However, TPM was ineffective in the pediatric group, possibly due to the null effect of capsaicin in TG CGRP expression in pediatric rats (**Figures 2D,F**). These results suggest that TPM is effective in peripheral inhibition of CGRP expression in TG with a better response at low dosage in adults (**Figures 2D,E**).

The capsaicin-induced depletion of dural CGRP was significantly rescued by TPM at 30 (*p* = 0.021) and 100 (*p* = 0.021), but not 10, mg/kg in the adult group (**Figures 2G,H**). In the pediatric group, the rescue of CGRP depletion is significant at 10 mg/kg (*p* = 0.029) and 30 (*p* = 0.043), but not obvious at 100 mg/kg (*p* = 0.08) (**Figures 2G,I**).

These results that TPM is effective in attenuating capsaicin-induced central and peripheral nociceptive responses in both age groups validate this animal model. It also suggests that the anti-migraine mechanism of TPM involves both central and peripheral inhibition of TGVS. A better response was observed at a high dose of TPM for the responsiveness at TCC and dura in contrast to a low dose for TG in adult rats. However, in the pediatric group, a better response was found at a high dose of TPM for the central responsiveness at TCC in contrast to a low dose for dura. The better responsiveness to TPM at a high dose centrally is similar in both age groups, but the trend of responsiveness is different peripherally in both ages. There may be a therapeutic window for a better response in both central and peripheral inhibition, such as 30 mg/kg in this model.

## Discussion

In this study, we found that VPA and TPM, two clinically effective antimigraine drugs, are effective in suppressing central (TCC) or/and peripheral (TGVS) migraine responses induced by capsaicin in both adult and pediatric rats. These results validate this animal model induced by *i.c.* capsaicin and reveal the age-dependent differences in the antimigraine mechanism of these two drugs. VPA suppressed c-Fos expression centrally, but not peripherally in the pediatric group, whereas induced peripheral, but not central, inhibition of the TGVS in adults. For TPM, the central suppression of c-Fos was significantly effective at higher dose (≥30 mg/kg) in both age groups. The peripheral inhibition of TGVS was significant in adults at the 3 doses in a dose-dependent way in contrast to pediatrics effective at a low dose. Interestingly, low-dose TPM is better than high-dose for peripheral suppression in adult TG and pediatric dura while high-dose TPM is better in central suppression at the TCC of both age groups. There may be an optimal therapeutic window for both central and peripheral inhibition, such as 30 mg/kg in this model. To the best of our knowledge, this is the first report to show the age differences in antimigraine mechanism of VPA and TPM.

### The Role of VPA in Migraine

Intravenous VPA is clinically effective for abortive therapy of migraine headaches in adults and children (Kinze et al., 2001; Ashrafi et al., 2005). It has been studied and used for migraine prevention with success (Linde et al., 2013). VPA has been theorized to modulate GABA receptors and sodium/calcium neuronal channels that inhibit the excitatory process seen in migraine headaches (Cutrer et al., 1997). In the same capsaicin-induced migraine model in adult guinea pigs, VPA suppressed the elevated c-Fos expression in the trigeminal nucleus caudalis (TNC) (Cutrer et al., 1995a) in a manner mediated via GABA_A_, but not GABA_B_, receptors (Cutrer and Moskowitz, 1996). In the present study, we investigated capsaicin-induced elevation of c-Fos immunoreactive neuronal number in the TCC that includes TNC and upper cervical spinal cord, and the central suppression by VPA in adult rats is not significant. Different extension of investigation and different species may cause the difference. A recent transcriptomic profile study by Kogelman et al. (2018) revealed distinct transcriptomic profiles between TNC and spinal dorsal horn tissues in adult rats. Although the actual mechanism remains unknown, it may contribute to different responses in the TNC and the TCC (which includes the TNC and upper cervical spinal cord) toward the VPA treatment in *i.c.* capsaicin-induced migraine model. As for the age-dependent differences, a better central inhibition of TCC in the pediatric than adult might result from a switch in the subunit composition of GABA_A_ receptor during postnatal development (Fritschy et al., 1994), suggesting that the existence of molecularly distinct immature and adult forms of GABA_A_ receptors may differently respond to VPA.

Among neuropeptides, CGRP is the most widely spread and abundant (Skofitsch and Jacobowitz, 1985; Ishida-Yamamoto and Tohyama, 1989). This peptide was found in ranging from approximately 35% to almost 50% neurons of the TG in rats and man, present in small-sized cells previously (Lazarov, 2002). In capsaicin-treated rats, we found CGRP was densely distributed in TG neurons (Fan et al., 2018). In contrast to the inhibition of TNC neuronal activation via GABA_A_ receptors, whether VPA treats migraine is mediated by CGRP remains unclear. VPA treatments had been shown to significantly reduce CGRP expression in dorsal root ganglia (DRG) and improve the generalized hyperalgesia resulting from induced endometriosis (Zhao et al., 2011). This study with capsaicin-induced migraine model first provides direct evidence that VPA inhibited CGRP expression in TG and reversed CGRP depletion in dura. However, whether this CGRP depressant effect is secondary to its potentiation of GABA effect warrants further investigation. As for the null responsiveness in TG toward VPA in capsaicin-treated pediatric rats, it may be attributed to the postnatal development of TG neuron and its surrounding satellite glial cells (SGC). Glial-neuron interaction in the TG is crucial in regulating CGRP expression and release in relation to migraine pathogenesis (Thalakoti et al., 2007). Hayasaki et al. (2006) reported that both neurons and SGCs were stained immunopositive for GABA, and GABA would be released to inhibit CGRP activity in TG neuron. Interestingly, Durham and Garrett (2010) demonstrated that in rat TG, the number of glial cells associated with an individual neuron increased more than 3-folds from P0–P56. Thus, it is implicated that the density of SGCs, and thus GABA producing units in TG tissue in the pediatric group may be less than that in adults. This may explain the null peripheral responsiveness in pediatric rats toward VPA, which is an inhibitor of GABA transaminase that degrades GABA and an activator of glutamic acid decarboxylase, a GABA synthesizing enzyme (Chapman et al., 1982; Rogawski and Loscher, 2004).

### The Role of TPM in Migraine

Topiramate is a neuromodulatory compound with stabilizing properties and has been demonstrated to modify several receptor- and voltage-gated ion channels, including Na^+^ channels, Ca^2+^ channels, α-amino-3-hydroxy-5-methylisoxazole-4-propionic acid (AMPA)/kainate receptors, and GABA_A_ receptors (White, 2005). The mechanism(s) of its antimigraine activity are unclear. We speculate that TPM might not inhibit c-Fos expression in TCC via GABA_A_ receptors in both ages because there are different trends between VPA and TPM in these two groups. It warrants further study to clarify the mechanism of central suppression by TPM in migraine.

In human, it was reported that intravenous administration of TPM attenuated the pain-related activity of parts of the thalamo-cortical network and increased functional coupling between the thalamus and several brain regions such as the bilateral precuneus, posterior cingulate cortex and secondary somatosensory cortex suggesting that TPM exhibits modulating effects on nociceptive processing in thalamo-cortical networks during trigeminal pain (Hebestreit and May, 2017). With the recent discovery of direct cortico-trigeminal inhibition from primary somatosensory cortex to TNC (Castro et al., 2017), and the existence of direct TNC innervation from secondary somatosensory cortex (Tashiro et al., 1983), it is speculated that TPM may activate that descending cortico-trigeminal inhibitory pathway, hence centrally suppressed the c-Fos expression in TCC of both pediatric and adult rats.

In the peripheral response, blocking peripheral GluR5 kainate receptors may contribute to its inhibition of neurogenic dural vasodilatation (Andreou et al., 2009). CGRP is involved in the anti-migraine mechanism of TPM has been shown in many *in vitro* studies (Storer and Goadsby, 2004; Akerman and Goadsby, 2005a, b; Durham et al., 2006). Here, we demonstrated TPM attenuated CGRP expression and release *in vivo*. The exact mechanism how TPM regulates CGRP release remains to be elucidated. It is interesting to notice that in adult rats, there is a dose-dependent response with a better response at low doses of TPM in both peripheral effects, the suppression of TG CGRP and the rescue of dural CGRP depletion, but it had a better effect at higher doses in central suppressing TCC neuronal activation. These results suggest an optimal dose window of TPM for migraine therapy, namely 30 mg/kg in this study, which is compatible with the clinical experience (Brandes et al., 2004; Silberstein et al., 2004; Pringsheim et al., 2012). We hypothesize that at least dual mechanisms may contribute to the antimigraine effects of TPM in central and peripheral locations and the role of the mechanisms may be different at high and low doses, respectively. Therefore, further investigation of the role of GABA_A_ receptor, kainate receptor, and L-type calcium channel is required in the future.

## Conclusion

In conclusion, both VPA and TPM are effective in central or/and peripheral suppression of capsaicin activation in both adult and pediatric rats. These results validate that this capsaicin-induced animal model can mimic migraine clinically. The antimigraine responsiveness to VPA and TPM are different in both pediatric and adult animals. VPA involved central suppression of TCC in the pediatric rat, in contrast to peripheral inhibition of TGVS in adult. TPM suppressed both central and peripheral responsiveness in both ages with an optimal therapeutic window. The exact mechanisms how c-Fos is suppressed by TPM and CGRP released is modulated by both drugs warrants further investigation The current findings may shed light on the clinical observations of varied responsiveness in pediatric migraineurs toward the treatment with VPA (Bakola et al., 2009; El-Chammas et al., 2013) or TPM (Unalp et al., 2008; Le et al., 2017).

### Limitation of the Study

The present study, for the first time demonstrates that VPA and TPM differentially exert their anti-migraine effect in pediatric and adult rat model of migraine induced by *i.c.* instillation of capsaicin, via central and peripheral responses of TGVS. This *in vivo* model is a useful tool to study the histopathological changes in migraine-associated areas such as TG, the TCC and the dura mater, and has been employed to evaluate the pharmacological potency of various anti-migraine agents, including clinically effective VPA and a sumatriptan analog (Cutrer and Moskowitz, 1996; Cutrer et al., 1999). However, there are some limitations to the present model. First, in the present study the animals were under anesthesia throughout the study protocol. Further studies will have to be conducted to examine the effects of VPA and TPM in migraine-associated behaviors such as allodynia, facial grimace and photophobia (Harris et al., 2017). Second, only male rats were used in the present study. Further studies will be conducted to investigate the presence of sexual difference, with association with age-difference, in responsiveness toward migraine therapy.

## Author Contributions

PH and P-CF designed and conducted the experiments, analyzed the data, and wrote the paper. ML and LC-C contributed to data interpretation and paper writing. P-HK contributed to results discussion and paper writing.

## Conflict of Interest Statement

The authors declare that the research was conducted in the absence of any commercial or financial relationships that could be construed as a potential conflict of interest.
